# Borderland Studies

**Published:** 1896-12

**Authors:** 


BORDERLAND STUDIES. Miscellaneous addresses and essays pertaining to medicine and the medical profession and their relations to general science and thought. By GEORGE M. GOULD, A. M., M. D., formerly editor of the *Medical News*. Price, $2. Philadelphia : P. Blakiston, Son & Co., 1012 Walnut street. 1896.

From the title of this work one would imagine it a treatise on psychiatry or a series of essays dealing with that class of patients commonly designated as borderland cases. On reading the preface, however, this notion is soon dispelled by the statement that as

"many of the essays contained in this volume are at present out of print it has been thought best to reproduce them in a single volume." To these essays the author has added several hitherto unpublished and a number of editorial articles from the *Medical News*, published during the author's editorship of that journal. The list of essays and articles is as follows : 1. Vivisection ; 2. Concerning medical language ; 3. The rôle of the maternal instinct in organic evolution ; 4. Life and its physical basis ; 5. Is medicine a science ? 6. The duty of the community to medical science ; 7. Charity : organisation and medicine ; 8. Hospitalism ; 9. The etiology, diagnosis and treatment of the prevalent epidemic of quackery, being the addresses delivered by the author in this city before the graduating class of the University of Buffalo, May 3, 1892, and which aroused considerable feeling among the homeopathic practitioners ; 10. The untrustworthiness of the lay press in medical matters ; 11. The disorganisation of medical science ; 12. Concerning specialism ; 13. Medicine and city noises ; 14. Medical aspects of life insurance ; 15. Foot-ball—in this paper the author makes some excellent points against this un-American form of athletics ; 16. Muscular development and use the conditions of health ; 17. Everybody's medical duty ; 18. The power of will in disease ; 19. The apotheosis of hysteria and whimsicality ; 20. Character ; 21. The modern Frankenstein ; 22. Dreams, sleep and consciousness ; 23. Human life under denied sensation ; 24. Immortality. These essays are all written in the author's forcible and earnest manner ; they make interesting and instructive reading.

